# Fiber-Optic Based Smart Textiles for Real-Time Monitoring of Breathing Rate

**DOI:** 10.3390/s20123408

**Published:** 2020-06-17

**Authors:** Aizhan Issatayeva, Aidana Beisenova, Daniele Tosi, Carlo Molardi

**Affiliations:** 1 Department of Civil and Environmental Engineering, Nazarbayev University, Nur-Sultan 010000, Kazakhstan; 2 Department of Electrical and Computer Engineering, Nazarbayev University, Nur-Sultan 010000, Kazakhstan; aidana.beisenova@nu.edu.kz (A.B.); daniele.tosi@nu.edu.kz (D.T.); carlo.molardi@nu.edu.kz (C.M.); 3 Laboratory of Biosensors and Bioinstruments, National Laboratory Astana, Nur-Sultan 010000, Kazakhstan

**Keywords:** breathing pattern monitoring, fiber optics, Fiber Bragg Grating (FBG) sensors, smart textile, respiration rate monitoring

## Abstract

Wearable light textiles are gaining widespread interest in application for measurement and monitoring of biophysical parameters. Fiber optic sensors, in particular Bragg Grating (FBG) sensors, can be a competitive method for monitoring of respiratory behavior for chest and abdomen regions since the sensors are able to convert physical movement into wavelength shift. This study aims to show the performance of elastic belts with integrated optical fibers during the breathing activities done by two volunteers. Additionally, the work aims to determine how the positions of the volunteers affect the breathing pattern detected by optical fibers. As a reference, commercial mobile application for sensing vibration is used. The obtained results show that the FBGs are able to detect chest and abdomen movements during breathing and consequently reconstruct the breathing pattern. The accuracy of the results varies for two volunteers but remains consistent.

## 1. Introduction

Wireless devices have pushed forward medical science to an advanced level in which people have access to a personalized drug delivery, a remote healthcare including simple diagnostics and data-logging operations outside of hospitals, and a continuous monitoring of biophysical parameters such as blood pressure, body temperature, breathing rate, etc. Low-cost miniature technologies help to prevent sudden infant death syndrome, heart-related diseases and provide minimal invasive continuous monitoring.

Several authors studied different techniques for designing smart textiles to measure breath pattern. For example, Skrzetuska and Wojciechowski [1] studied the ability of T-shirts equipped with a printed respiratory rate sensor to monitor the breathing pattern of two volunteers and the influence of the environmental humidity and temperature on the output of the sensor. The authors have tried several configurations of printed sensor and identified the most optimal shape and size of the sensor. The breathing of volunteers were monitored during physical activities and rest. The sensing technology were able to identify breaths but the external climate conditions were found to have an effect on the accuracy of the results.

Joyashiki and Wada [2] proposed to monitor breathing pattern by a body-conducted sound sensor placed on the neck. The performance of the sensor was compared with two other sensors, namely air-coupled microphone and acceleration sensor. A data analysis technique based on signal processing was developed. The authors came to the conclusion that body-conducted sensor performs better in four different types of the experiments. Schatz et al. [3] studied the application of five different types of depth sensors for breathing rate monitoring and usage of this data for the sleep apnea identification. All of the sensors output were compared with the reference sensor and two of the five sensors have been found appropriate for sleep apnea determination. Jayarathna et al. [4] proposed to use carbon-balck elastomer sensor embedded on the textile to measure respiratory rate, cardiac cycle and body position during sleep. The authors achieved a precision of 88.9%–100% in respiratory rate monitoring. Klum et al. [5] proposed to use multimodal patch stethoscope and monitor breathing rate based on the electrocardiogram and phonocardiogram derived approaches. Neural network has been applied for the data analysis and the setup was able to correctly identify most of the breaths. Roudjane et al. [6] designed a respiratory rate monitoring sensor based on a spiral silver-doped hollow-core fiber antenna, a Bluetooth transceiver, an energy harvesting module and Raspberry Pi base module. The sensor was embedded into the T-shirt and the setup was able to detect breathing patterns of four volunteers.

Since sensors network devices such as Arduino boards, wireless transceivers which are based on networking protocols like Wi-Fi or Zigbee are considered bulky and inconvenient for daily usage, several authors have considered fiber optic devices for this application [7]. Optical fibers are considered as fire-safe, immune to electromagnetic radiation and compact in terms of size [8]. Due to a broad variety of advantages of the optical fibers, they have been gradually used for the smart textile applications as a sensing and monitoring technology [9].

For example, Leal-Junior et al. [10] presented smart textiles achieved by equipping the wearable with a polymer optical fiber sensor for simultaneous measurements of the breath and heart rates. A signal processing analysis was developed in order to obtain the required heart and breathing rates and filter the person’s body movements. The authors achieved accurate results: the error for the heart beat is below 4 beats per minute and the error for the respiratory rate is lower than 2 breaths per minute.

The same authors [11] also presented the smart walker system, which consists of a 3D printed handle with an oximetry sensor and smart clothes equipped with the polymer optical fiber sensors. The smart walker system is capable of measuring oxygen saturation, gait cadence, breathing and heart rates with an accuracy of 99%. The system is proposed to be applied in the remote healthcare and robotics applications.

Gil et al. [12] developed a real-time wireless body sensor network which simultaneously measures physical parameters such as electroencephalography to record a brain activity, photoplethysmography to record a pulse, electrocardiography to detect a heart activity and respiration rate. The system has four major units: multi-sensing module, digitalizing module, power model and monitoring-analyzing system. The network obtains the parameters from a human body, represents outputs in a frequency domain by using Fast Fourier Transform and finally sends them to a smartphone or a PC through a Bluetooth module. The authors succeeded in synchronizing four inputs with a current of 104.1 mA (STD is ± 18.3 mA) and a data rate of 16,384 bits which is 5.9 frames per second [12]. On the other hand, it is stated in [13,14] that standard methods of respiration rate monitoring based on electronics cannot be used during daily activities because they require supporting tools which are bulky.

Massaroni et al. claim that smart textiles are obtaining widespread attention for the continuous monitoring of respiratory rate and sensing units such as fibers, yarns and threads with piezoresistive properties can be embedded into elastic belts [15]. The latter one has been investigated in [15], specifically, six piezoresistive elements have been proposed to monitor a respiratory physiology in the regions of abdominal rib cage, pulmonary rib cage and abdomen. The assessment of a performance of the system during quiet breathing and tachypnea indicates its capability to monitor chest movements in a wide range of frequency. Results show that for quiet breathing the difference of average frequencies between reference and sensing units is lower than 1% while for tachypnea the values are always lower than 4%. By definition, smart textiles have an ability to sense parameters based on the type of sensors integrated in it. In this context, significant development in integration of fiber optic sensors (FOS) into textiles has been driven by a great number of researchers. The use of FOS is reasonable due to its characteristics such as fast response, high sensitivity, chemical and biological inertness [16]. Additionally, FOS do not generate a heat and are immune to electrical discharge in comparison with electrical devices.

Koyama et al. incorporated a hetero-core optical fiber into a textile for monitoring heartbeat and respiration of a healthy adult in a sitting position [17]. The smart textile was sewn into the inner side of a wool cardigan in the region of left chest. The hetero-core optical fiber changes the light intensity of the transmitted light when hetero- core portion is induced to a bending. During the breathing, since the textile is enclosed between a wool cardigan and an adult’s body, the fiber experiences a load which is proportional to peak frequencies in the obtained spectrum. The radius of the curvature is 6 mm and the value of the applied force should not exceed 25 N in order to avoid fiber damage [18].

Another optical fiber device which is suitable for a measurement of body parameters, including the blood pressure and stroke volume [19,20], the temperature change in thermal ablation [21,22], is Fiber Bragg Grating (FBG) sensor. The use of FBG for the respiratory rate detection purposes has been demonstrated in [23,24,25]. The recent works about the integration of FBG into a textile have been done by European project OFSETH (optical fiber sensors embedded into technical textile for healthcare). The FBG allows to discriminate a strain by locating sensor belts in thoracic and abdominal regions in MR environment. Additionally, a single FBG embedded in a PVC laminate shows a sensitivity of 0.8 pm/μϵ [26,27]. The sensor measures cardiac frequency and breathing rate by showing strain peaks.

The aim of this paper is to contribute to the research area related to a wearable textile technology for a real-time continuous monitoring of the biophysical parameters in order to achieve a personalized health control. This work makes use of of two arrays of five FBG sensors embedded into elastic belts, located on the chest and on the abdomen, for the respiratory rate monitoring. Each array is composed by 5 FBGs for a total of 10 points of measurement. Specifically, the proposed design aims to convert breathing movement, detected in chest and abdomen regions of volunteers, to strain values. Moreover, the good number of redundant sensing points, located in different positions, is used to better reconstruct the breathing pattern also when the breath reading is disturbed by different body movements. A specific algorithm, using a diversity technique, has been purposely developed to exploit the information coming from the 10 sensing points. By using a mobile app VibSensor, which is sensitive to vibrations, as a reference unit, the influence of volunteers’ position on the breathing rate is identified and compared with the readings coming from our system.

## 2. Methodology

The aim of the experiments is to study the feasibility of the FBGs arrays for breathing pattern monitoring application. The breathing pattern is measured at the two locations of the body (abdomen and chest) by two arrays of 5 FBGs.This allows to apply a diversity technique, which is used in communication systems to achieve better accuracy of detection by combining the outputs from several different sensing points [28]. The breathing pattern has been measured in four different positions of the volunteers (sitting, lying, staying or running). Two volunteers have participated in the initial experiments: 23-years old woman, height 165 cm, weight 52 kg and 24-years old man, height 171 cm, weight 72 kg. The volunteers wore T-shirts with two specially designed belts located on the abdomen and chest regions. They have been asked to breath for 23 seconds in different positions: staying, sitting, lying and running. The results of the the strain change detected by FBGs have been compared with the output of a reference sensor, which is a mobile application for acceleration and vibration measurement. The mobile phone with the application has been attached to the upper belt.

**Figure 1:**
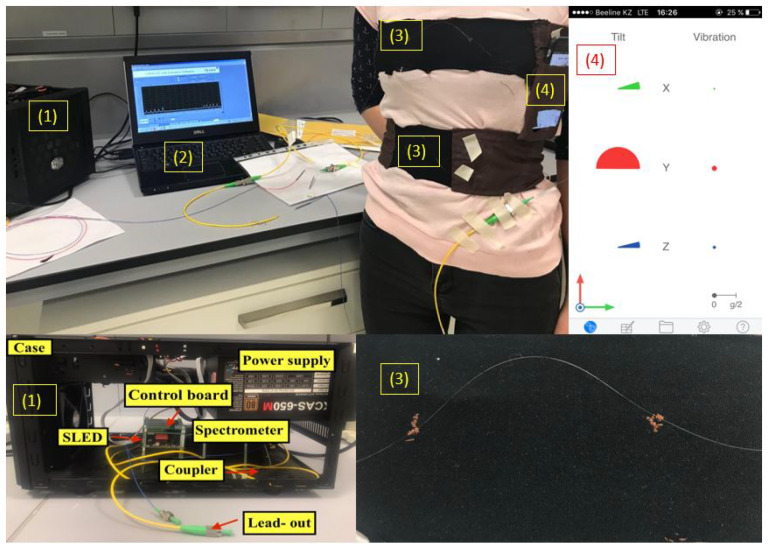
Experimental setup: (1) I-MON interrogator, (2) PC with evaluation software, (3) T-shirt with belts equipped with arrays of 5 FBGs, (4) VibSensor mobile application.

The experimental setup, which is illustrated in Figure 1, consists of the (1) I-MON interrogator connected to (2) PC with evaluation software, (3) T-shirt with two belts each equipped with an array of 5 FBGs, (4) mobile phone with VibSensor application.

### 2.1. Interrogation System

**Figure 2:**
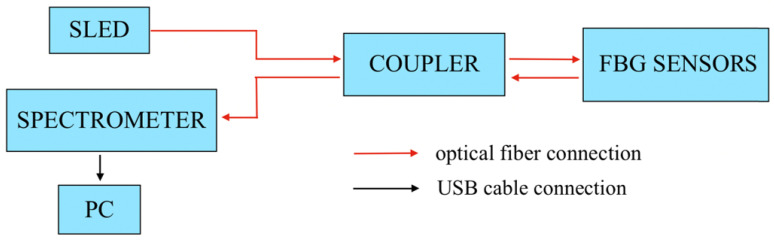
Schematic view of the system: light from SLED travels to the FBG sensors and reflects back to the spectrometer through a coupler for decoding and measurement in PC.

The data acquisition was performed by I-MON 512 USB (Ibsen Photonics, Sweden) interrogator, shown in Figure 1. The schematics of the interrogator is illustrated in Figure 2 illustrates its schematics. The interrogator consists of a superluminescent LED (SLED, Exalos EXS, 1520–1600 nm, 10 mW), a control board, which is responsible for stabilizing of driving current and operative temperature, a power supply, a spectrometer and a 50/50 coupler which delivers a light to the optical fiber with FBG sensors. I-MON 512 USB has an evaluation software which displays and saves the raw data. The wavelength fits resolution of the system is less than 0.5 pm and the measurement frequency is 3000 Hz [29]. The software provides the wavelength spectrum as an output. MATLAB script has been developed in order to convert the wavelength spectrum to the strain change pattern. The size of the interrogator used in this experiment is 37 cm × 24 cm × 19 cm. It is our future goal to reduce the interrogator size in order to make it portable. The preliminary experiments show encouraging results.

### 2.2. Fiber Bragg Grating Sensors

In the experiments, two arrays of 5 FBGs sensors manufactured by FBGS International have been used. Each FBG sensor has a length of 5 mm and the center to center distance between two neighboring FBGs is 10 mm. The wavelengths of the first FBGs array are in the range from 1540 nm to 1548 nm and the space between the peaks of the neighboring FBGs is 2 nm. The FBGs of the second array are located in the range from 1570 nm to 1578 nm with the same spacing of 2 nm between the two consequent peaks.

When a spectrum of light propagates through the fiber a specific wavelength, called Bragg wavelength, is reflected whereas the remaining part of the light is transmitted [30]. When an external temperature or strain are induced, FBGs react accordingly by causing a proportional shift of the reflected Bragg wavelengths. The Bragg wavelength is represented by the following equation [31]:

(1) $$\lambda_{B}=2n_{eff}\Lambda .$$

where Λ is the periodicity of the gratings and $n_{eff}$ is the effective refractive index of the fundamental mode.

**Figure 3:**
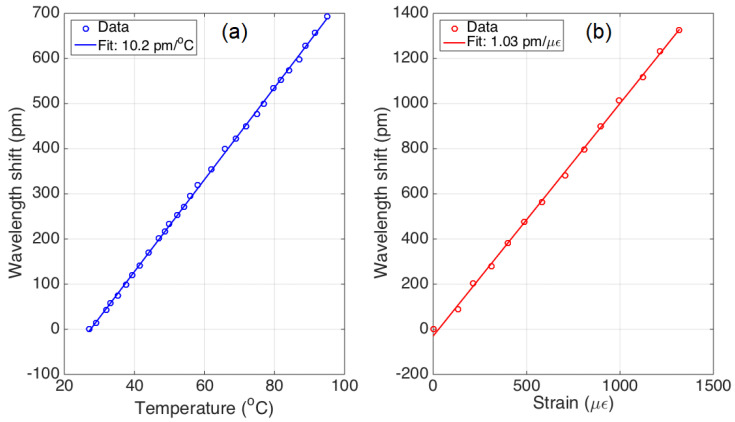
Experimental characterization of temperature (**a**) and strain coefficients (**b**).

The sensitivity coefficients for temperature and strain of the FBGs, used in this work, have been experimentally characterized, the found values are: $k_{T}$ = 10.2 pm/${}^{\circ }$C for temperature, and $k_{\epsilon }$ = 1.03 pm/μϵ for strain. The characterization curves are shown in Figure 3. Since the FBGs arrays have been manufactured with a draw tower inscribing the FBBGs in a standard SMF-28 fiber, the characteristics of each FBGs are substantially identical, in terms of thermal and strain properties. The experimental found coefficients matched the values declared by the manufacturer.

The temperature and strain coefficients are used to express the wavelength shift, according to the following equation [32]:

(2) $$\Delta \lambda_{B}=\lambda_{B_{0}}+k_{\epsilon }\Delta \epsilon +k_{T}\Delta T$$

where $\lambda_{B_{0}}$ is the Bragg wavelength when an optical fiber in a reference position, Δϵ is the strain change and ΔT is the temperature shift.

**Figure 4:**
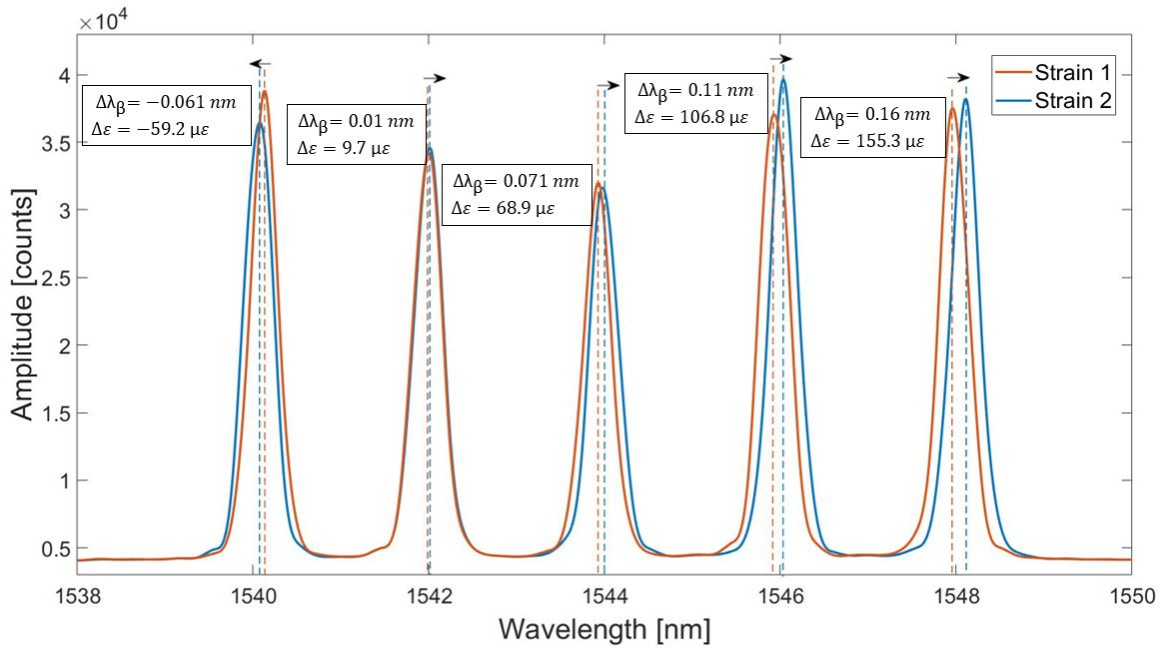
Reflection spectrum of the 5 FBGs array exposed to different strains (strain 1 and strain 2). The arrows shows the direction of the wavelength shifts for each FBG due to the strain applied to the array.

The fiber with five FBGs has been bent in order to achieve strain change. Figure 4 shows change of the spectrum of the FBGs array due to this applied strain change (from strain 1 to strain 2). This resulted in the wavelength shifts detected by each FBGs differently. The first left FBG has experienced compression, so its wavelength shift is negative. The other four FBGs have been under tension and detected the positive, but different strains. According to Figure 4, the second FBG from the left has experienced a small strain change although the fiber has been bent. This shows that some of the points on the fiber can be stretched more than others at the same moment. Therefore, the multi-point sensing is used in the experiments in order to cover more area and increase the accuracy of the breathing detection. The information from all FBGs can be analysed collectively in order to obtain the correct pattern.

### 2.3. Belt Design and Fibers Fixation

As can be seen in Figure 1, there are two belts embedded into the T-shirt, one on the chest region and another one on the abdomen. The belts consist of two parts: brown fabric is inelastic, and the black fabric is very elastic. The belts need to fit around the chest and the abdomen. The FBGs are placed on the elastic part because when the person breathes, the elastic part stretches and this elongation is detected by FBGs. As a result, by this design the FBGs are able to monitor the movements of the chest and the abdomen.

**Figure 5:**
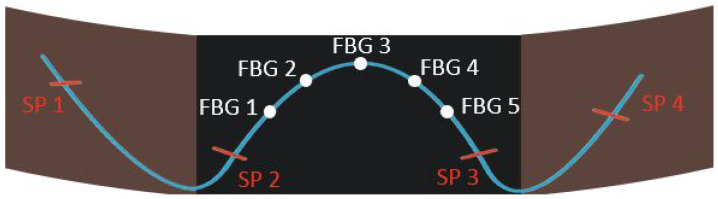
Fiber sewing points (SP 1, 2, 3 and 4) and FBGs position (FBG 1, 2, 3, 4 and 5) on the belts.

Figure 5 shows the fixation of the array of 5 FBGs on the elastic part of the material. As it can be seen, the fiber is fixed at four points and the FBGs are not fixed in order to have an opportunity to move. The order of FBGs start from the left to the right: from FBG 1 to FBG 5 for the chest region and from FBG 6 to FBG 10 for the abdomen region.

### 2.4. Reference Sensors

To check the validity of the optical fiber sensors for the breathing pattern monitoring a reliable reference sensor is required. One of the options is to use available mobile applications which measure acceleration or vibrations. The accuracy of 12 mobile applications for vibration measurements have been studied by Cahil et al., who compared their performance with the tri-axial accelerometer [33]. According to this study, the VibSensor application has shown a good precision, so it is selected as a reference in the experiments. The application is available on the App Store and provides the graphs of the acceleration change over time in three directions (x—left and right, y—up and down, z—back and forth). Mobile phone with the application has been positioned on the chest as shown in Figure 1. Although, the application does not directly measure the breathing pattern, the expansion of the chest causes movement of the mobile phone, at least, in one direction. Therefore, the acceleration pattern of the mobile phone can provide the information about breathing pattern.

### 2.5. Algorithm for the Breathing Pattern Reconstruction

As a result of the experiments, I-MON interrogator has registered the wavelength per time of each of 10 FBGs. The algorithm, that reconstructs the breathing pattern from the wavelength pattern obtained from I-MON, consists of the following steps:

(1) Convert wavelength pattern per time to the strain change by using Equation (2). Since temperature in this experiment is constant, ΔT is 0. Then, the equation can be rearranged to find strain:

(3) $$\Delta \epsilon =\frac{\left(\Delta \lambda_{B}-\lambda_{B0}\right)}{k_{\epsilon }}.$$

**Figure 6:**
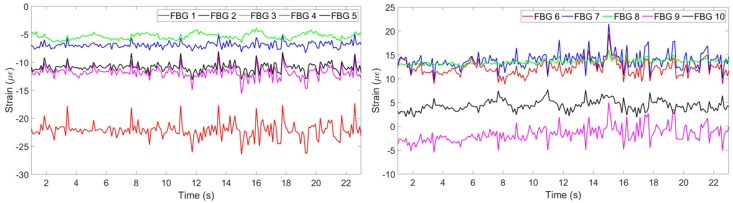
Strain pattern detected by 5 FBGs on the chest (**left**) and by 5 FBGs on the abdomen (**right**).

Figure 6 shows the strain change per time detected by each FBG.

**Figure 7:**
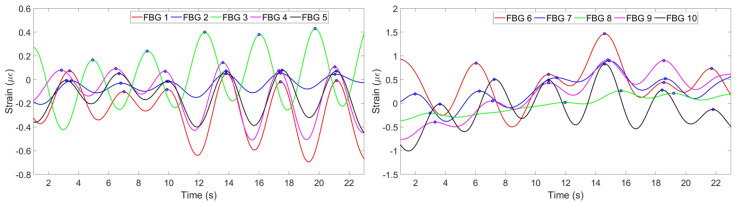
Strain pattern after application of filter detected by 5 FBGs on the chest (**left**) and by 5 FBGs on the abdomen (**right**).

(2) The strain change detected by FBGs is a result of not only breathing, but all the movements of the chest and abdomen including heart beat and motions of a person. Therefore, the strain has to be filtered in order to obtain the components responsible for breathing only. This has been done by converting the strain pattern to frequency domain by Fast Fourier Transform. People’s normal breathing rate is about 20 breaths per minute [34] (0.33 Hz), so everything higher than this frequency has been filtered. Then the signal has been converted back to time domain and the strain patterns shown on Figure 7 have been obtained.

(3) According to Figure 7, FBGs 1, 2, 4 and 5 on the chest region have similar patterns and have detected 6 peaks: on the 3rd, 7th, 10th, 14th, 17th and 21st seconds. Third FBG also has similiar pattern but with opposite sign because while other FBGs experience tension, this FBG feels tension. FBGs 6, 7, 9 and 10 on the abdomen region have also detected 6 peaks correctly, but FBG 8 has unclear pattern. It can be seen, that using this information eight FBGs out of ten can be used for reconstruction, third FBG on the chest needs to be reflected to satisfy the others pattern. One FBG out of ten in this case has given inaccurate result being unable to correctly identify the breathing pattern.

The algorithm is aimed at finding the most common pattern, reflecting opposite strains and neglecting discrepant patterns. This is achieved by firstly identifying positive and negative peaks of all FBGs. If at least 6 FBGs out of ten detected either positive or negative peak at approximately same time, this peak is considered as correct. The strain pattern of FBGs with correct negative peaks are multiplied by −1. FBGs which have less than 30% correctly detected peaks are assumed to produce wrong pattern and is neglected by multiplying its strain change by 0.

**Figure 8:**
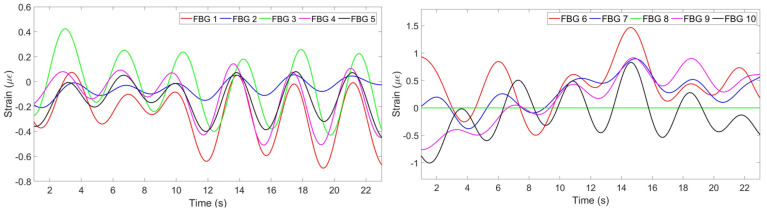
Strain pattern after reflecting opposite patterns and neglecting wrong patterns detected by 5 FBGs on the chest (**left**) and by 5 FBGs on the abdomen (**right**).

Figure 8 illustrates the strain patterns of all FBGs after the implementation of the algorithm. It can be seen that the third FBG on the chest region has been reflected, so now its pattern corresponds to the general trend. The eighth FBG on the abdomen region has been multiplied by 0 in order not to be considered in the further analysis.

**Figure 9:**
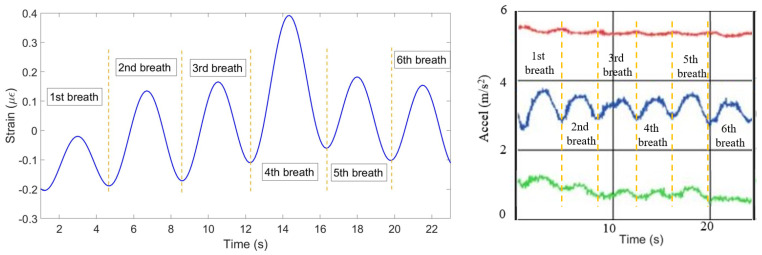
Breathing pattern reconstructed based on the 10 FBGs strain patterns (**left**) and obtained from the reference sensor (**right**).

(4) The mean of all ten patterns was calculated and the single breathing pattern shown on Figure 9 (left) has been achieved. The algorithm helped to identify 6 breaths at the following seconds: 0–4, 4–8, 8–12, 2–16, 16–20 and 20–23. Figure 9 (right) shows the plot obtained from the reference sensor, which has also detected 6 breaths during 23 s.

## 3. Results and Discussion

The same algorithm has been applied for both volunteers in staying, sitting, lying and running positions.

### 3.1. Staying Position

**Figure 10:**
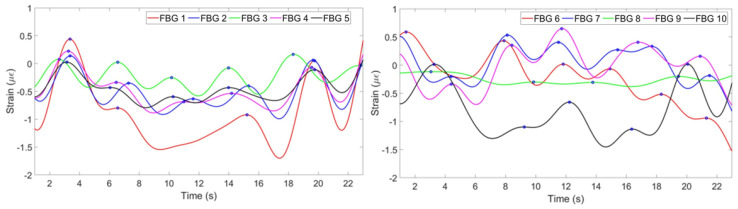
Breathing pattern for the first volunteer in the staying position detected by FBGs on the chest (**left**) and abdomen (**right**) regions.

**Figure 11:**
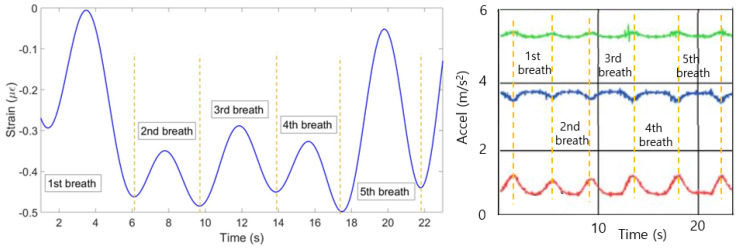
Breathing pattern for the first volunteer in the staying position reconstructed based on the 10 FBGs strain patterns (**left**) and obtained from the reference sensor (**right**).

Figure 10 illustrates strain patterns of ten FBGs during the experiment with the first volunteer in the staying position. As it can be seen on the Figure, most of the FBGs have identified five peaks almost at the same time. The exception is FBF 8, which has identified only four peaks at the different from the majority time periods. Therefore, the algorithm has filtered the eighth FBG in order the final plot not to be influenced by this incorrect pattern. The mean of the rest nine FBGs has resulted in the breathing pattern shown in Figure 11 (left). The setup has detected five breaths which satisfies the reference sensor‘s output given in Figure 11 (right).

**Figure 12:**
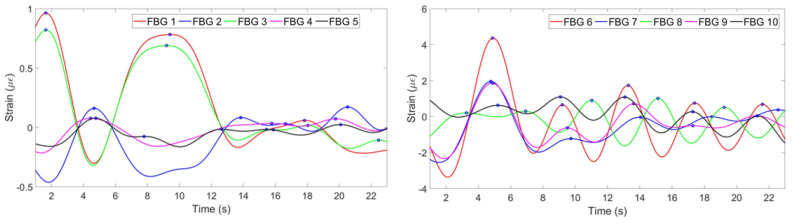
Breathing pattern for the second volunteer in the staying position detected by: FBGs on the chest (**left**) and abdomen regions (**right**).

**Figure 13:**
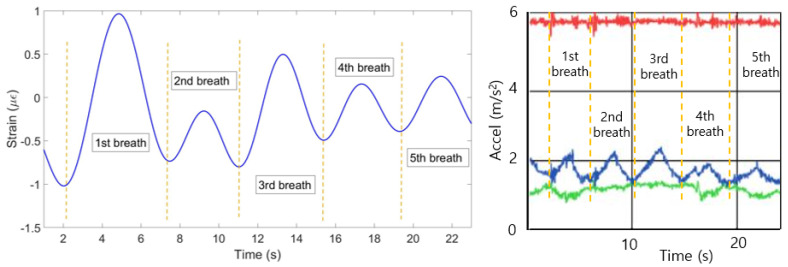
Breathing pattern for the second volunteer in the staying position reconstructed based on the 10 FBGs strain patterns (**left**) and obtained from the reference sensor (**right**).

According to the Figure 12, which depicts the results of the experiments with the second participant in the staying position, the first and the third FBGs on the chest and the eighth FBG on the abdomen have negative strain patterns. The algorithm has reflected the patterns of those three FBGs. The result of the mean of the ten FBGs is shown in Figure 13 (left). The breathing pattern consists of five breaths which is in agreement with the pattern produced by the reference sensor in Figure 13 (right).

### 3.2. Sitting Position

**Figure 14:**
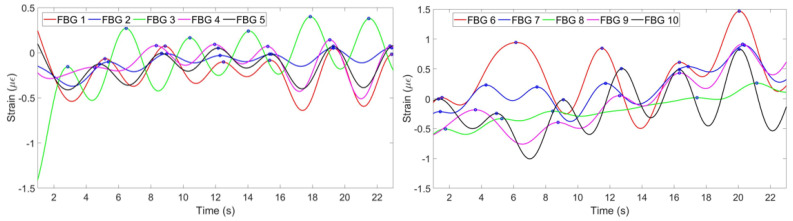
Breathing pattern for the first volunteer in the sitting position detected by FBGs on the chest (**left**) and abdomen regions (**right**).

**Figure 15:**
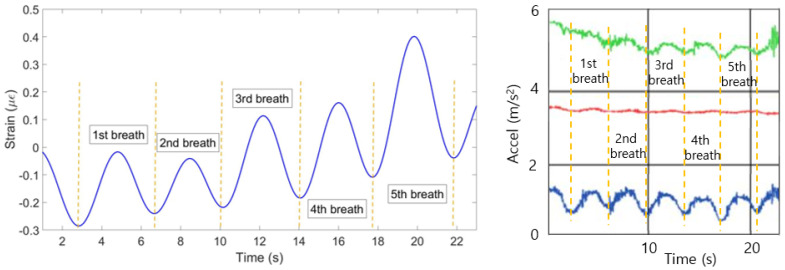
Breathing pattern for the first volunteer in the sitting position reconstructed based on the 10 FBGs strain patterns (**left**) and obtained from the reference sensor (**right**).

The result of the experiment with the first participant in the sitting position is illustrated in Figure 14. It can be seen, that the third FBG on the chest has opposite to others strain pattern, while the eighth FBG on the abdomen has unclear pattern. Therefore, the third FBG is reflected and the eighth FBG is zeroed by the algorithm. The combined respiratory pattern of ten FBGs is given in Figure 15 (left). Five breaths have been detected by FBGs setup, as well as by the reference sensor shown in Figure 15 (right).

**Figure 16:**
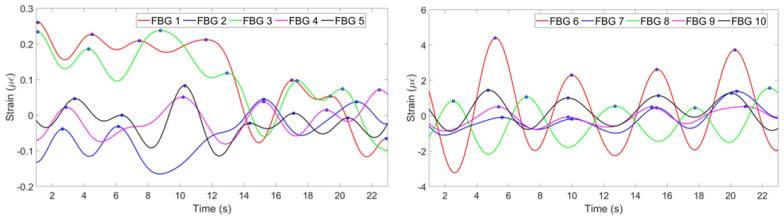
Breathing pattern for the second volunteer in the sitting position detected by: FBGs on the chest (**left**) and abdomen regions (**right**).

**Figure 17:**
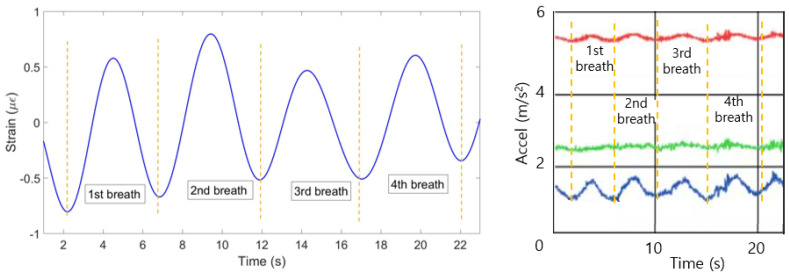
Breathing pattern for the second volunteer in the sitting position reconstructed based on the 10 FBGs strain patterns (**left**) and obtained from the reference sensor (**right**).

Respiratory rate patterns of the FBGs during the experiments with the second participant in the sitting position is shown in Figure 16. As it can be seen, the first and the third FBGs on the chest and the eighth FBG on the abdomen have negative patterns. Those three strain patterns have been reflected by the algorithm and the mean of the 10 FBGs resulted in the respiratory rate pattern illustrated in Figure 17 (left). The setup identified four breaths which correspond to the output of the reference sensor shown in Figure 17 (right).

### 3.3. Lying Position

**Figure 18:**
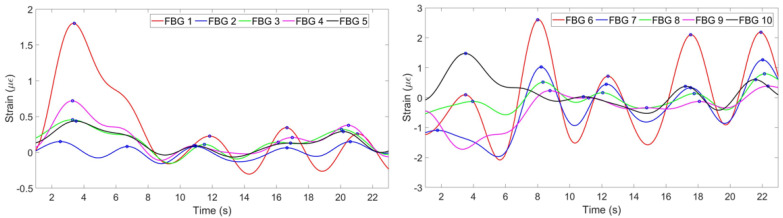
Breathing pattern for the first volunteer in the lying position detected by: FBGs on the chest (**left**) and abdomen regions (**right**).

**Figure 19:**
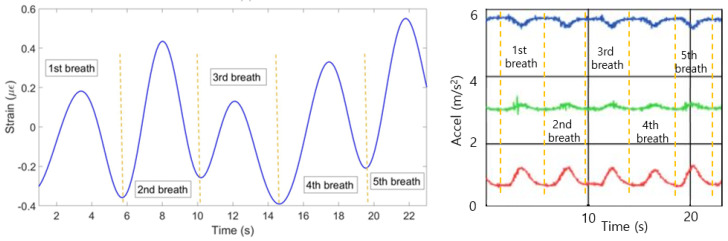
Breathing pattern for the first volunteer in the lying position reconstructed based on the 10 FBGs strain patterns (**left**) and obtained from the reference sensor (**right**).

According to Figure 18, which illustrates the strain pattern for the first volunteer in the lying position, all of the FBGs have peaks at the same time periods. The algorithm has not detected any negative or wrong patterns, therefore the plots have not changed. The mean of ten FBGs gives the breathing pattern depicted in Figure 19 (left). Both FBGs setup and the reference sensor have detected five breaths.

**Figure 20:**
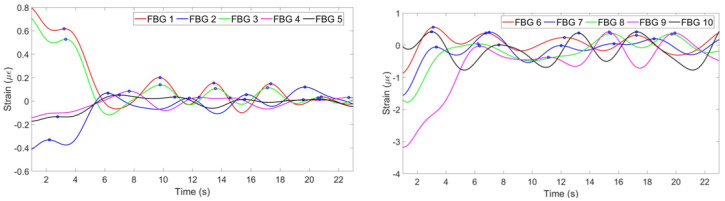
Breathing pattern for the second volunteer in the lying position detected by: FBGs on the chest (**left**) and abdomen regions (**right**).

**Figure 21:**
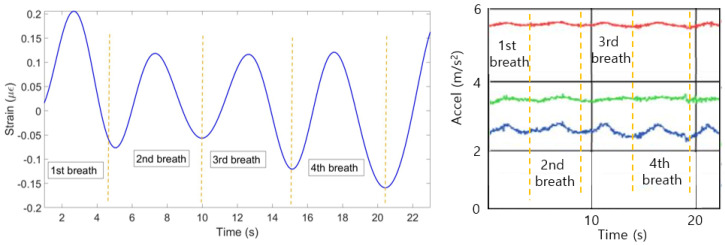
Breathing pattern for the second volunteer in the lying position reconstructed based on the 10 FBGs strain patterns (**left**) and obtained from the reference sensor (**right**).

The results of the experiments with the second participant in the lying position are illustrated in Figure 20. The first and the third FBGs on the chest and the eighth and the ninth FBGs on the abdomen show negative patterns. Therefore they have been reflected by the algorithm. Figure 21 (left) shows that the mean of the resultant FBGs patterns represents a pattern which consists of four breaths. This corresponds with the number of breaths detected by the reference sensor illustrated in Figure 21 (right).

### 3.4. Running Position

**Figure 22:**
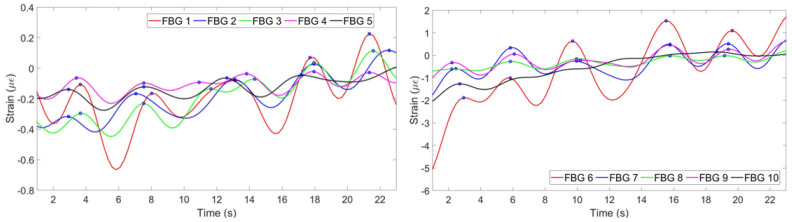
Breathing pattern for the first volunteer in the running position detected by: FBGs on the chest (**left**) and abdomen regions (**right**).

**Figure 23:**
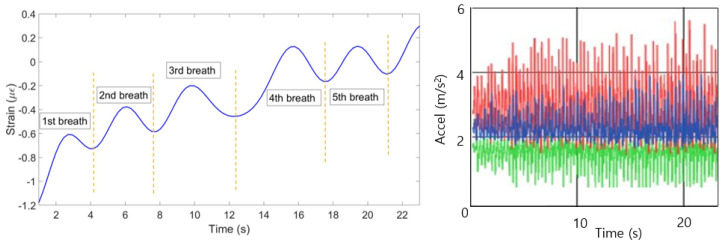
Breathing pattern for the first volunteer in the running position reconstructed based on the 10 FBGs strain patterns (**left**) and obtained from the reference sensor (**right**).

Strain patterns for the experiment with the first participant in the running state is shown in Figure 22. Out of ten FBGs only the last one on the abdomen region gives wrong pattern and is zeroed by the algorithm. Figure 23 (left) illustrates that the combination of ten FBGs, which have identified five breaths. As it can be seen in Figure 23 (right), the reference sensor in this case is not able to measure breathing pattern due to the movement of the participant which interferes the breathing pattern.

**Figure 24:**
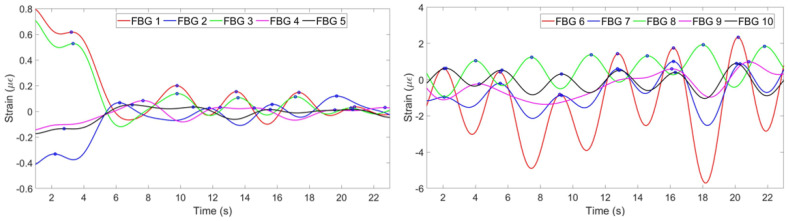
Breathing pattern for the second volunteer in the running position detected by: FBGs on the chest (**left**) and abdomen regions (**right**).

**Figure 25:**
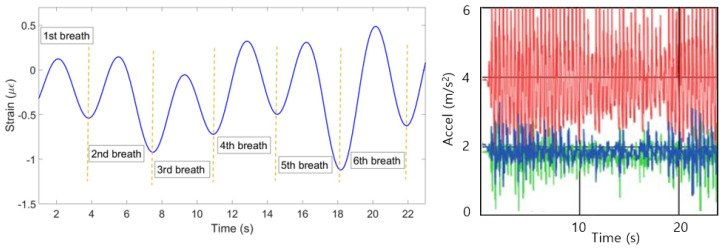
Breathing pattern for the second volunteer in the running position reconstructed based on the 10 FBGs strain patterns (**left**) and obtained from the reference sensor (**right**).

The result of the experiment with the second participant in the running state is given in Figure 24. According to the Figure, the second, the forth and the fifth FBGs on the chest and the eighth FBG on the abdomen have opposite to the majority patterns. Therefore, they have been reflected by the algorithm. According to Figure 25 (left), the setup has detected six breaths. The reference sensor, as in previous case, was unable to monitor the breathing pattern.

## 4. Conclusions

The experiments have shown that the FBGs have a promising potential in the smart textile application for the respiratory pattern monitoring. According to the analysis, some of the FBGs can fail to detect the correct pattern because of the location on the non-stretching point at that time. In order to avoid reliance on a single FBG, multi-point sensing is proposed (in this case 5 FBGs on each region, but it can be increased or decreased in the future). Both the chest and the abdomen regions have shown to be effective in identification of breathing patterns. Due to the fact that some FBGs experience strain while others are compressed, the patterns can have opposite signs. Therefore, in order to be able to combine the outputs from all sensors, the negative patterns have to be identified and reflected. This and the depreciation of the wrong patterns have been achieved using the simple algorithm. The proposed technology can be applied for people in ordinary life because it is able to monitor breath in all possible human positions (staying, sitting, lying and running). Further improvement of the results can be achieved by testing other setup configurations: tighter adjustment of belts, decreased length of inelastic part of the belt, another configuration of FBGs.
